# Factors Affecting the Introduction of New Vaccines to Poor Nations: A Comparative Study of the *Haemophilus influenzae* Type B and Hepatitis B Vaccines

**DOI:** 10.1371/journal.pone.0013802

**Published:** 2010-11-02

**Authors:** Aharona Glatman-Freedman, Mary-Louise Cohen, Katherine A. Nichols, Robert F. Porges, Ivy Rayos Saludes, Kevin Steffens, Victor G. Rodwin, David W. Britt

**Affiliations:** 1 Global Public Health Master's Program, New York University, New York, New York, United States of America; 2 Department of Family and Community Medicine, New York Medical College, Valhalla, New York, United States of America; 3 Department of Pediatrics, Albert Einstein College of Medicine, Bronx, New York, United States of America; 4 Department of Obstetrics and Gynecology, New York University, New York, New York, United States of America; 5 Robert F. Wagner School of Public Service, New York University, New York, New York, United States of America; 6 Department of Health and Sports Sciences, University of Louisville, Louisville, Kentucky, United States of America; The George Washington University Medical Center, United States of America

## Abstract

**Background:**

A major effort to introduce new vaccines into poor nations of the world was initiated in recent years with the help of the GAVI alliance. The first vaccines introduced have been the *Haemophilus influenzae* type B (Hib) and the hepatitis B (Hep B) vaccines. The introduction of these vaccines during the first phase of GAVI's operations demonstrated considerable variability. We set out to study the factors affecting the introduction of these vaccines. The African Region (AFRO), where new vaccines were introduced to a substantial number of countries during the first phase of GAVI's funding, was selected for this study.

**Methodology/Principal Findings:**

GAVI-eligible AFRO countries with a population of 0.5 million or more were included in the study. Countries were analyzed and compared for new vaccine introduction, healthcare indicators, financial indicators related to healthcare and country-level Governance Indicators, using One Way ANOVA, correlation analysis and Qualitative Comparative Analysis (QCA). Introduction of new vaccines into AFRO nations was associated primarily with high country-level Governance Indicator scores. The use of individual Governance Indicator scores, as well as a combined Governance Indicator score we developed, demonstrated similar results.

**Conclusions/Significance:**

Our study results indicate that good country-level governance is an imperative pre-requisite for the successful early introduction of new vaccines into poor African nations. Enhanced support measures may be required to effectively introduce new vaccines to countries with low governance scores. The combined governance score we developed may thus constitute a useful tool for helping philanthropic organizations make decisions regarding the type of support needed by different countries to achieve success.

## Introduction

Childhood mortality continues to be a major global public health problem. In 2005, approximately 9.7 million children under the age of 5 died worldwide [1]. The United Nations Millennium Developmental Goals (MDGs) delineated in 2002, express a need for action through goal number 4, aiming for a two-third reduction in childhood mortality from 1990 to 2015 (www.un.org/millenniumgoals/pdf/mdg2007.pdf). Many childhood deaths occurring in poor nations result from diseases and conditions which are easily preventable by vaccines that are readily available in developed nations. Of the worldwide estimated 2.5 million deaths from vaccine preventable diseases among children younger than 5 years of age, 87% occur in poor regions of the world [2]. Furthermore, it is estimated that approximately 162 million Disability-Adjusted Life Years (DALYs) are lost worldwide from vaccine-preventable diseases, more than 90% of them in low income countries [3]. Until recently, funds for supply and administration of vaccines in poor nations have been scarce and inconsistent [4]–[6].

In 2000, a new global alliance for vaccine and immunization, the GAVI Alliance (formerly known as the Global Alliance for Vaccines and Immunizations), was established in an effort to end vaccine inequity between rich and poor nations [5]. The GAVI Alliance is a public-private partnership whose partners include UN agencies, the World Health Organization (WHO), the World Bank, public health institutions, donor and recipient countries, the Bill and Melinda Gates Foundation, pharmaceutical manufacturers, and other members of the philanthropic and financial community (www.gavialliance.org/about/in_partnership/index.php). GAVI's initial objectives were to provide the basic vaccines (Polio, Diphtheria, Pertussis, Tetanus, Measles and BCG) as well as new and underused vaccines – particularly *Haemophilus influenzae* type b (Hib), Hepatitis B (HepB) and Yellow Fever, to children in developing nations (www.unicef.org/chinese/immunization/files/immunize_every_child.pdf; http://www.gavialliance.org/performance/global_results/index.php). GAVI's funding has been available to any nation with a Gross National Income (GNI) per capita under $1,000 (www.gavialliance.org/support/who/index.php) following an application review by independent experts (www.gavialliance.org/support/how/index.php).

During its first phase of operation (2000–2005), GAVI provided support for two new vaccines that have been readily available to children in developing countries, the Hib and HepB vaccines (a third vaccine against Yellow Fever is used only in Africa and South America).

Hib is the most common cause of meningitis and a leading cause of pneumonia in un-immunized infants and children under the age of 5 years [reviewed in [7]; [8]. HepB virus can cause acute and chronic Hepatitis, cirrhosis of the liver and hepatocellular carcinoma; it can be transmitted at birth, through intimate or sexual contacts and via needle sticks [9]. Vaccines are the most effective preventive measure against both pathogens [9]; [10]. Despite the proven effectiveness of the Hib vaccine, its introduction into immunization programs of developing nations has been slow as compared with the HepB vaccine. By the end of 2005, fewer than 20 million children worldwide received a full 3 doses series of Hib vaccine as compared to more than 95 million children that had received all 3 required doses of HepB (www.gavialliance.org/performance/index.php). During the first phase of GAVI's operation, the number of GAVI-eligible countries using Hib vaccine increased from 3 to 19, while HepB vaccine usage increased from 17 to 57. (www.gavialliance.org/resources/15brd_HepBHibYF_zuber_28Apr05.ppt); [11].

The purpose of this study was to investigate the conditions which have been conducive to the successful introduction of the Hib and HepB vaccines by GAVI into poor nations during its first phase of operations. Recent experiences suggest that for healthcare projects to succeed in poor countries, governments are central to the delivery of services on a regional, national or global level, even in the context of operations led by non-governmental organizations or the business sector [12]. Furthermore, governments in poor countries were in some cases chief funders of public healthcare efforts [12]. We thus examined systematically the association of new vaccine introduction with different elements of the healthcare, finance and governance context, characterizing the nations in our sample of GAVI-eligible African Nations.

## Methods

### Countries

GAVI-eligible nations of the WHO African region (AFRO) with a population size of 0.5 million or more were included in the study (www.who.int/about/regions/afro/en/index.html). Only the island of Sao Tome and Principe (population size 157,000) was excluded (www.who.int/about/regions/afro/en/index.html). A total of 35 countries were studied.

### Data Collection

Vaccination data were obtained from WHO/UNICEF reports (www.who.int/vaccines/globalsummary/immunization/countryprofileresult.cfm). Years of HepB and Hib vaccine initiation were recorded for each country.

Country population sizes and Healthcare Indicators statistics were obtained from the WHO database (www.who.int/whosis/en/index.html). The healthcare indicators evaluated for each country were: Life expectancy for males, life expectancy for females, number of doctors per 1,000 people and number of nurses per 1,000 people. The financial indicators related to healthcare evaluated were: Total Healthcare Expenditure per Capita (THECAP), Government Healthcare Expenditure per Capita (GHECAP) and Total Healthcare Expenditure as percent of the GDP (HEGDP).

Country-level Governance Indicators scores for each country were obtained from the World Bank database (http://info.worldbank.org/governance/wgi/index.asp). These included: Political Stability (PS), Government Effectiveness (GE), Rule of Law (Law), Regulatory Quality (Reg), Control of Corruption (Corr), and Voice and Accountability (VA). The scores were provided as percentile rank with higher values indicating higher performance.

The indicator values for 2005 were collected first, and if statistically significant differences were found between the country groups, indicator values were then collected and evaluated for the years 1995 to 2005.

### Statistical Analysis

Statistical analysis was done using SPSS version 15.0 for PC. Mean, median and standard error were determined for each of the continuous variables. Comparisons between different groups of countries were done using One Way ANOVA. A *p* – value of <0.05 was considered statistically significant.

Correlation coefficients were calculated to evaluate the relationship between variables using Pearson's and Point Biserial tests. Reliability was determined using Chronbach's alpha.

Qualitative Comparative Analysis (QCA) was used to examine the alternative combinations of factors that are conducive to the success or failure of new vaccine introduction. QCA was performed using fsQCA software.

## Results

### New vaccine programs in AFRO nations

New vaccine introduction data revealed that GAVI-eligible African countries could be divided into 3 distinct groups based on the status of new vaccine introduction or use during the first phase of GAVI's operation (2000–2005). The groups were defined as follows (Table 1):

- Countries in which both Hib and Hepatitis B vaccine were introduced.
- Countries in which Hepatitis B vaccine, but not Hib vaccine, was introduced.
- Countries in which neither Hib nor Hepatitis B vaccine were introduced.

**Table 1:** New vaccine introduction to GAVI-eligible AFRO countries.*

Group IHib and HepB			Group IIHepB		Group IIINeither vaccine
Country	Year	Year	Country	Year	Country
	HepB	Hib		HepB	
Benin	2002	2005	Cameroon	2005	Angola
Burkina Faso #	2006	2006	Comoros	2001	Chad
Burundi	2004	2004	Cote D'Ivoire	2001	CAR
Gambia &	1990	1998	Eritrea	2002	Congo
Ghana	2002	2002	Lesotho	2001	Congo DR
Kenya	2002	2002	Madagascar	2003	Ethiopia
Malawi	2002	2002	Mauritania	2005	Guinea
Mali	2003	2005	Mozambique	2001	Guinea-Bissau
Rwanda	2002	2002	Nigeria	2005	Liberia
Senegal	2004	2005	Tanzania	2002	Niger
Uganda	2002	2002	Zimbabwe	1999	Sierra-Leone
Zambia	2005	2004			Togo

*Countries divided according to new vaccine (Hib and Hepatitis B) introduction by 2005 and WHO/Unicef coverage reporting by 2006 (first vaccine coverage reporting year is provided).

# The application for HepB and Hib vaccines submitted by Burkina Faso was approved in October 2004 (www.gavialliance.org/resources/Info_Update_December2004.pdf).

& Gambia started HepB and Hib vaccination program on its own. It was approved for GAVI's support for these vaccines in 2001 (www.gavialliance.org/performance/country_results/index.php?countID=23).

### Country population size

The mean country population size was calculated for each country group. No statistical differences were found between the mean country sizes of the 3 groups.

### Healthcare Indicators

The 3 country groups were compared for differences and similarities in male and female life expectancy and number of doctors and nurses per 1,000 people available by 2005, using One Way ANOVA. No statistically significant differences were found between the 3 vaccination country groups.

### Financial Indicators related to Healthcare

**Figure 1:**
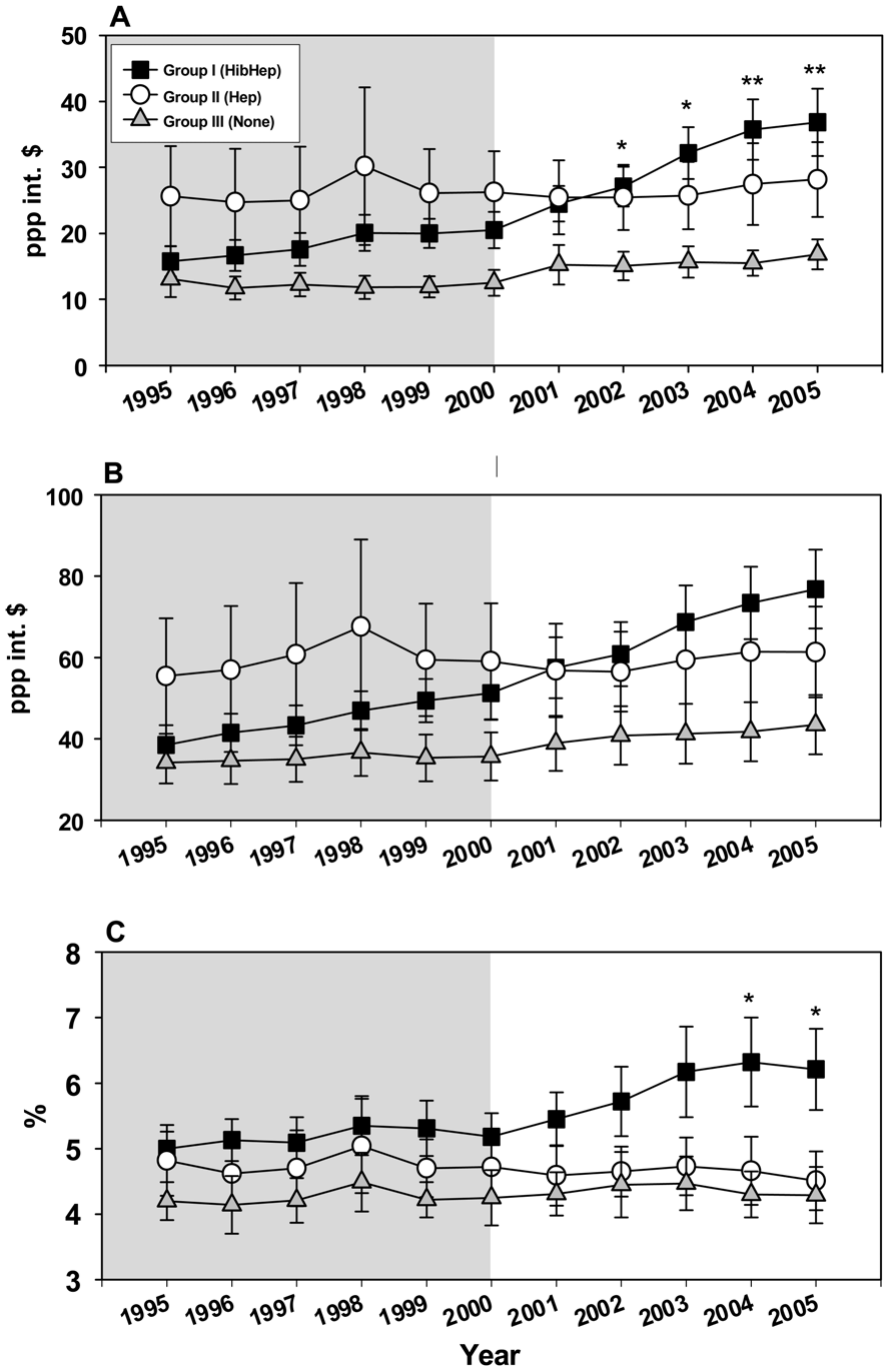
Financial healthcare indicators during the pre-GAVI and the first phase of GAVI's funding. (A) GHECAP; (B) THECAP; (C) HEGDP. Symbols represent means and error bars represent standard error of the mean. Grey plot background highlights the Pre-GAVI years, white plot background highlights the GAVI funding years. * *p* value<0.05, ** *p* value<0.01. Placement of (*) above square symbols denotes a statistically significant difference between country groups I and III.

The 3 county groups were compared for differences and similarities in Total Healthcare Expenditure per Capita (THECAP), Government Healthcare Expenditure per Capita (GHECAP) and Total Healthcare Expenditure as percent of the GDP (HEGDP) using One Way ANOVA. Mean, standard deviation and standard error of the mean of country group expenditure values were first evaluated for 2005, and statistically significant differences were found between the groups for Total Healthcare Expenditure as percent of the GDP (HEGDP) and Government Healthcare Expenditure per Capita (GHECAP) (Figures 1A, 1C). Data were then collected for each country for the years 1995–2005. Data for Total Healthcare Expenditure per Capita (THECAP) were collected as well to evaluate pattern of expenditure. The data summary is presented in Figure 1. For all 3 indicators the means for group III were the lowest throughout the period studied. The mean values for group II was higher and remained relatively consistent throughout the period studied for all 3 indicators. The mean values for group I of all indicators increased gradually with the highest expenditure growth rate occurring after 2000 (the beginning of GAVI's funding period). For group I, GHECAP and THECAP expenditure values were similar to those of group III in the early pre-GAVI years, and by the end of the GAVI's first phase of funding, their values were the highest among the 3 groups (Figures 1A, 1B).

For GHECAP, statistically significant differences were found between group I and III for the years 2002, 2003 (*p* value<0.05), 2004 and 2005 (*p* value<0.01) (Figure 1A). For HEGDP, the differences between groups I and III reached statistical significance in the years 2004 and 2005 (p value<0.05) (Figure 1C). For THECAP, the pattern of expenditure was similar to that of GHECAP, but no statistically significant differences were demonstrated between the groups (Figure 1B).

### Country-level Governance Indicators

Political Stability, Government Effectiveness, Regulatory Quality, Rule of Law, Control of Corruption and Voice and Accountability scores (expressed as percentile rank) were collected for each country. Country group means were then calculated for each of the indicators. After determining that the country group mean for 2005 for each indicator showed statistically significant differences, scores were evaluated for the years 1996–2005 for each country (with the exception of 1999 and 2001 for which scores were not available).

**Figure 2:**
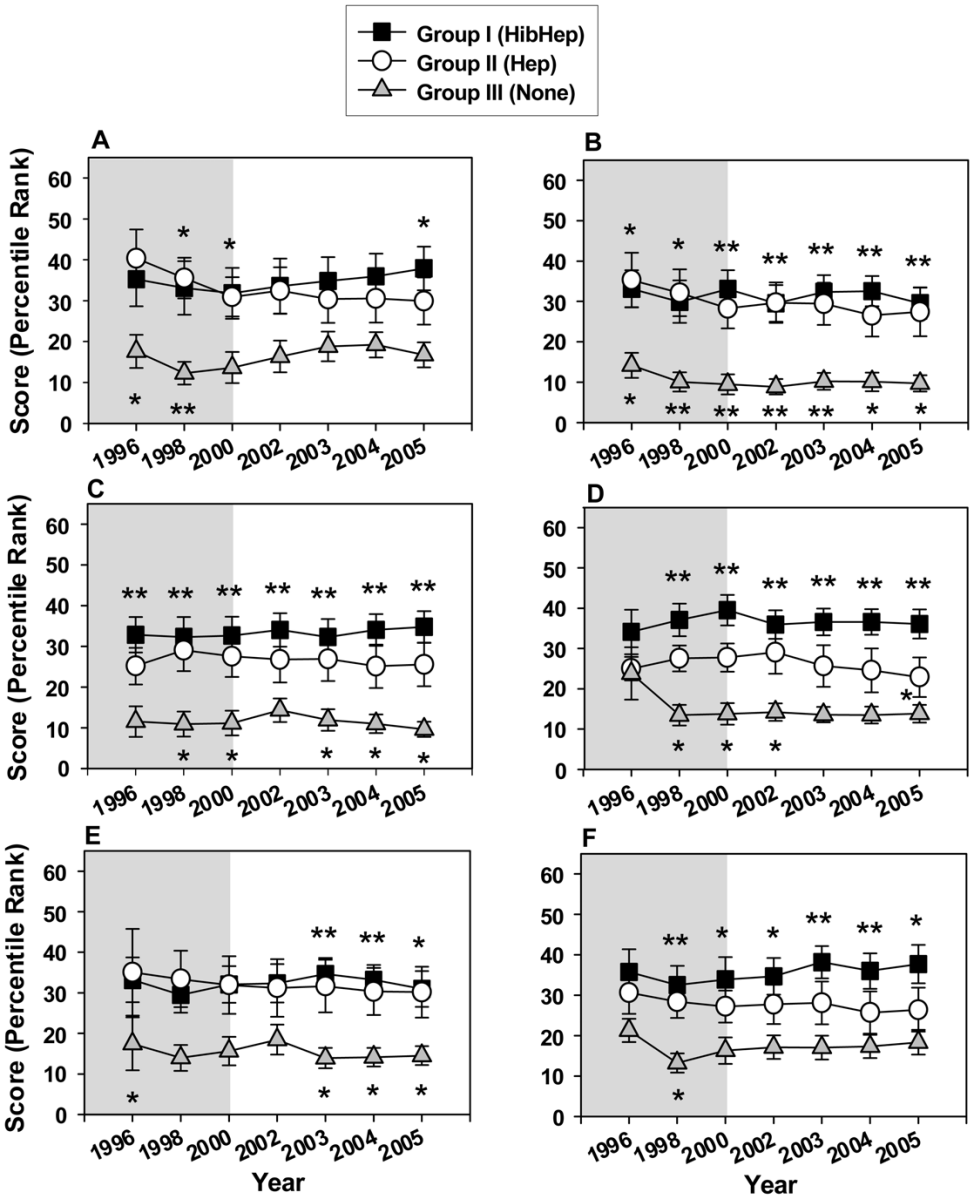
Governance Indicators for the pre-GAVI and the first phase of GAVI's funding. (A) Political stability; (B) Government effectiveness; (C) Rule of Law; (D) Quality control; (E) Control of corruption; (F) Voice and accountability. Symbols represent means and error bars represent standard error of the mean. Grey plot background highlights the Pre-GAVI years, white plot background highlights the GAVI funding years. * *p* value<0.05, ** *p* value<0.01. Placement of (*) above square symbols denotes a statistically significant difference between country groups I and III. Placement of (*) under triangle symbols denotes a statistically significant difference between country groups II and III. Placement of (*) under circle symbols denotes a statistically significant difference between country groups I and II.

Overall, the mean scores were highest for group I and lowest for group III (Figure 2). Multiple comparisons analysis demonstrated that statistically significant differences were found between groups I and III for all indicators (Figure 2A–F). Statistically significant differences between groups II and III were also found for all indicators, however, less frequently (Figure 2). Statistically significant differences were found between group I and group II only for Regulatory Control in 2005 (Figure 2D).

### Correlation and reliability studies

Given the similar patterns of the various Governance Indicators scores for the country groups (figure 2), we examined the correlation between them for the years 1996 to 2005. A strong positive correlation was found among all the Governance Indicators (Table 2), with a statistically significant correlation coefficient (*p-value*<0.01) for all of them. Analysis of correlation between Governance Indicator means and country groups (Table 2) demonstrated a statistically significant relationship (*p* value≤0.01). Reliability analysis performed using all the Governance Indicators for all the countries, demonstrated a Cronbach's Alpha score of 0.902.

**Table 2:** Correlation between Governance indicator means for the years 1996–2005 and country groups.

		CountryGroup	PSmean	GEmean	LawMean	RegMean	CorrMean	VAmean
**Country Group**	Pearson Correlation	1	−.434(**)	−.570(**)	−.595(**)	−.651(**)	−.425(*)	−.517(**)
	Sig. (2-tailed)		.009	.000	.000	.000	.011	.001
	N	35	35	35	35	35	35	35
**PSmean** #	Pearson Correlation	−.434(**)	1	.662(**)	.798(**)	.608(**)	.621(**)	.783(**)
	Sig. (2-tailed)	.009		.000	.000	.000	.000	.000
	N	35	35	35	35	35	35	35
**GEmean** #	Pearson Correlation	−.570(**)	.662(**)	1	.831(**)	.823(**)	.697(**)	.720(**)
	Sig. (2-tailed)	.000	.000		.000	.000	.000	.000
	N	35	35	35	35	35	35	35
**LawMean** #	Pearson Correlation	−.595(**)	.798(**)	.831(**)	1	.742(**)	.791(**)	.704(**)
	Sig. (2-tailed)	.000	.000	.000		.000	.000	.000
	N	35	35	35	35	35	35	35
**RegMean** #	Pearson Correlation	−.651(**)	.608(**)	.823(**)	.742(**)	1	.566(**)	.699(**)
	Sig. (2-tailed)	.000	.000	.000	.000		.000	.000
	N	35	35	35	35	35	35	35
**CorrMean** #	Pearson Correlation	−.425(*)	.621(**)	.697(**)	.791(**)	.566(**)	1	.443(**)
	Sig. (2-tailed)	.011	.000	.000	.000	.000		.008
	N	35	35	35	35	35	35	35
**VAmeanV**	Pearson Correlation	−.517(**)	.783(**)	.720(**)	.704(**)	.699(**)	.443(**)	1
	Sig. (2-tailed)	.001	.000	.000	.000	.000	.008	
	N	35	35	35	35	35	35	35

**Correlation is significant at the 0.01 level (2-tailed).

*Correlation is significant at the 0.05 level (2-tailed).

# PS – Political Stability, GE – Government Effectiveness, Law – Rule of LAW, Reg – Regulatory Quality, Corr – Control of Corruption, VA – Voice and accountability.

### Development of a combined governance scoring system

**Figure 3:**
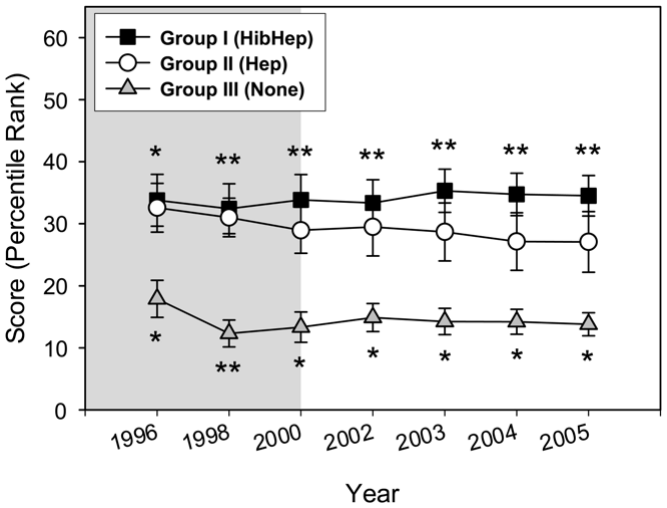
Combined Governance Indicator scores for the pre-GAVI and the first phase of GAVI's funding. Symbols represent means and error bars represent standard error of the mean. Grey plot background highlights the Pre-GAVI years, white plot background highlights the GAVI funding years. * *p* value<0.05, ** *p* value<0.01. Placement of (*) above square symbols denotes a statistically significant difference between country groups I and III. Placement of (*) under triangle symbols denotes a statistically significant difference between country groups II and III.

Given the high correlation and reliability scores for the Governance Indicators, we set out to develop a single governance score that will reflect the contribution of each indicator. A Combined Governance Score, consisting of the average score for all the Governance Indicators of each country for each year, was calculated. Combined Governance Score means were then calculated for each country group for each year in the period studied. These scores, shown in Figure 3, demonstrated similar pattern to that of the individual Governance Indicators, with the highest scores for group I and the lowest for group III. Differences between group I and III, were statistically significant with a *p value* of <0.01 for each year evaluated, except for 1996 when *p value* was found to be <0.05 (Figure 3). The differences between group II and III were statistically significant with a *p value* of <0.05 for all the years studied. No statistically significant differences were found between group I and II (Figure 3).

### Prediction of success in the introduction of new vaccines

To complement the group comparisons across time, we used QCA. Introduced by Ragin in 1987 [13], QCA is an analytic technique which utilizes Boolean algebra for the purpose of making multiple comparisons of various combinations of conditions, to determine which combinations of conditions are most favorable to a certain outcome. Thus, it is especially adept at examining the alternative combinations of contextual elements that are conducive to programs' success or failure. QCA is intended for use in studies with small to intermediate N [13]. In the present study, QCA helps draw attention to the alternative combinations of healthcare finance and governance conditions that are associated with the successful introductions of new vaccines.

The variables used for this analysis were the Combined Governance Score, HEGDP and GHECAP, for the pre-GAVI period, for each country (thus, the pre-GAVI means for the Combined Governance Score were obtained by using the values for 1996 and 1998 for each country, and the pre-GAVI mean for HEGDP and GHECAP were obtained by using the values for 1995–1999 for each country). The median value of each mean indicator score was then used to define high and low values. High values were assigned the number 1, low values were assigned the number 0, and these were then termed ‘conditions’ following standard QCA practice. Table 3 demonstrates all the possible combinations for the tested conditions. In addition, it demonstrates the number of countries with each of the specific condition combination, the number of countries in which a specific combination of conditions was associated with the introduction of at least one new vaccine, and the proportion of the countries that the specific combination of conditions was associated with the introduction of at least one new vaccine (defined as Consistency).

**Table 3:** Prediction of introduction of at least one new vaccine based on indicator combination.

IndicatorIndicatorCombination	Combined Governance Score	HEGDP	GHECAP	Number of countries with condition combination	No. of countries with the condition combination that introduced at least one new vaccine	Condition Combination Consistency	Success in the introduction of at least one new vaccine
**1**	1	1	1	9	8	0.89	1
**2**	0	0	1	6	3	0.5	0
**3**	0	1	0	6	2	0.33	0
**4**	0	0	0	5	1	0.2	0
**5**	1	0	0	3	3	1.0	1
**6**	1	0	1	3	3	1.0	1
**7**	1	1	0	3	3	1.0	1
**8**	0	1	1	0	0	0	0

If a combination of conditions was associated with a proportion of at least 0.75 of the countries being successful in having at least one new vaccine introduced, that combination was coded as a 1. If a proportion of less than 0.75 of the countries was associated with that outcome, the combination was considered as not successful in having at least one new vaccine introduced and was coded as 0.

Of the 18 countries with condition combinations that included high Combined Governance Score, 17 introduced at least one new vaccine (Table 3). In comparison, of 18 countries with condition combination that included either a high HEGDP or GHECAP score, 13 and 14 countries respectively introduced at least one new vaccine (Table 3). Thus, these results demonstrate that countries with high Combined Governance Score (alone or in combination with high HEGDP or GHECAP) for the pre-GAVI years were more likely to introduce at least one new vaccine than countries with high HEGDP and/or GHECAP score (alone or in combination with Combined Governance Score).

Overall, high Combined Governance Score of a country was found to be the only condition associated with most cases of success in terms of introducing at least one new vaccine. Furthermore, this association with success occurred independently of the presence of high HEGDP or GHECAP score in the combination. As a result, the Combined Governance Score can be declared both necessary and sufficient for predicting the introduction of at least one new vaccine, with raw and unique solution coverage of 0.74 and solution consistency of 0.94. Thus, these results indicate that the Combined Governance Score by itself correctly predicted 74% of the countries that were successful in introducing at least one new vaccine (17 of the 23 countries belonging to groups I and II). The results further indicate that the Combined Governance Score demonstrated an average of 94% consistency (17 of the 18 countries with high Combined Governance Score). No good QCA solutions were found for predicting the introduction of two new vaccines or distinguishing between the introduction of one or two new vaccines.

## Discussion

The GAVI Alliance's initiative to introduce new vaccines into developing countries is of utmost importance for the health of children worldwide. The Alliance's role in providing the financial resources for this purpose is crucial, especially given the high costs of such vaccines. The full Hib and HepB vaccine series, for example, costs substantially more per child for a series of 3 vaccines, as compared to the battery of basic vaccines (Diphtheria, Tetanus, Pertussis, Polio, Measles, Tetanus and BCG) [14]. While the cost for the basic battery of vaccines has been US $1 per child, the addition of Hib and HepB vaccines has raised the cost to US $7–13 per child (www.who.int/mediacentre/factsheets/fs288/en/index.html).

The GAVI alliance's approach is unique in that it seeks to provide financial support for vaccines while empowering nations to become eventually self-sufficient in supporting their vaccination programs (www.who.int/immunization_financing/analyses/fsp/process/en/). To do so, GAVI initially required that countries receiving GAVI's support, outline in their applications their plans to finance the vaccines costs in the future, and commit to prepare a comprehensive Financial Sustainability Plan (www.who.int/immunization_financing/analyses/fsp/process/en/). However, to achieve sustainable success, it is crucial to identify the factors that contribute or hinder progress. Examining the first phase of GAVI's funding, we found that GAVI-eligible AFRO countries differed with respect to the introduction of new vaccines into their immunization programs. While some countries introduced both the Hib and HepB vaccines (Group I), other countries introduced only the Hep B vaccine (Group II). A third group of countries did not introduce either vaccine (Table 1).

An initial analysis looking at the end of GAVI's first phase of operation (2005) suggested that higher financial indicator scores of GHECAP and HEGDP and higher Governance Indicator scores were associated with the introduction of both the Hib and Hep B vaccines into countries' immunization programs (Figure 1). A more detailed analysis of the period from 1995 to 2005 (Figure 1) demonstrated that while means of Governance Indicators scores for group I (both Hib and Hep B vaccines introduced) remained high and relatively stable throughout the pre-GAVI and the first phase of GAVI's funding periods (Figure 2), the mean GHECAP and HEGDP values for country group I changed throughout these years. Starting at low values in 1995, followed by a gradual increase (Figure 1), they surpassed the values for both group II and III by 2002. It is important to note that the largest increase in the financial healthcare scores of group I occurred between the years 2000 and 2005 (Figure 1), coinciding with the first phase of GAVI's operations. This sharp increase in the financial healthcare values of group I countries also coincided with the overall rapid increase in developmental assistance for health (DAH) for low income countries during these years [15]. Additional research is required to understand the role and effect of GAVI's funding in these financial indicator increases of group I countries.

The stable pattern of governance scores for the 3 country groups before and during GAVI's first phase of funding, and the association of higher governance scores with the introduction of both HepB and Hib vaccines (country group I), solidly support the strength of governance as an important factor in the ability of countries to support healthcare initiatives, including the introduction of new vaccines. These patterns, coupled with the QCA analysis results, strongly indicate that governance is a stronger predictor for the introduction of new vaccines as compared to healthcare financial expenditure.

It is interesting to note that overall, the countries that belong to group II, which have similar governance scores to those of group I, did not demonstrate increase in financial healthcare indicator values during the first phase of GAVI's funding. This observation is particularly interesting given the fact that the differences in governance scores between group I and II were not statistically significant. Although it is possible that these small differences in governance scores between groups I and II (with group II scores being slightly lower than those of group I) were associated with the inability of countries belonging to group II to increase the financial healthcare expenditure and to introduce the Hib vaccine, our data do not support such a conclusion. Furthermore, according to the QCA analysis, no good solutions were found to distinguish between countries that introduced one or two new vaccines. Thus, these results suggest that the lack of introduction of a second new vaccine (Hib) may be due to governmental decisions, lack of funds, specific infrastructural issues or indefensible grant application for GAVI's Hib funds, rather than lack of governmental execution abilities. It is also possible that these differences are due to lack of sufficient country awareness for the role of Hib in causing mortality and morbidity in countries belonging to group II. However, recent global estimates demonstrating that most Hib-related deaths have occurred in developing nations in Africa and Asia [8], justify the need to introduce and expand the Hib vaccine usage in these countries.

Overall, our results indicate that country-level governance is the single most important factor in determining the ability of poor African nations to introduce new vaccines. Good governance offers an obvious advantage for a country's ability to move forward with new healthcare initiatives like the introduction of a new vaccine. New efforts require the commitment and attention of leadership at the top governmental levels who must obtain and commit funds over multiple years. In nations that are struggling financially, political stability and good governance are often important factors in attracting foreign aid [16]. In this regard, good country-level governance was previously shown to have a considerable impact on investments in developing countries made by for-profit healthcare service providers and large multinational pharmaceutical and biotechnology corporations [17].

In addition to funding, the introduction of new vaccines requires adequate infrastructure elements such as sufficient cold chain capacity, ability to reach remote locations, safe disposal of needles and syringes, as well as sufficient numbers of adequately trained personnel [18]. Lack or scarcity of these elements constitute significant barriers to the adoption of new vaccines [18]. Country-level governance may have a crucial effect on both the initiation and mobilization of these important elements of vaccine programs.

Combining Governance Indicators into a single index has the advantage of providing one measure of governance that will take into account all the indicators. Although overall, we found a correlation between the various indicators of governance, some countries received substantially different scores for different Governance Indicators. Thus, a combined Governance score which takes into account all the components of governance, without the need to evaluate each one of them separately, provides a user-friendly measure of governance.

Our results indicate that a scoring system that takes into account all Governance Indicators (Figure 3) may constitute an effective quantitative method to predict the ability of poor nations in Africa to introduce new vaccines. There is a great need for quality quantitative tools to support decision making in healthcare philanthropy, and efforts to develop such tools are thus far at their infancy (http://aspe.hhs.gov/hsp/09/philnpart/chapter5.shtml). During its first phase of operations, GAVI strongly encouraged nations to apply for new vaccines. GAVI has been using a rigorous approach to evaluating, awarding and monitoring its grants, while attempting to consider individual countries' priorities, and promoting planning and country ownership. However, an independent evaluation of the first phase of GAVI found that it did not use a formal framework in directing its decision making process regarding approval of funding (www.gavialliance.org/resources/5._GAVI_Phase_1_Evaluation_Executive_Summary.pdf). Thus, the association of new vaccines introduction with high country-level governance scores reflects a phenomenon that is independent of a specific framework. Our findings could provide a basis for designing a framework and/or criteria that will guide the evaluation process and the support required for introducing new vaccines into nations with different levels of governance scores.

Overall, our findings suggest that for a new vaccine programs to succeed, special considerations and criteria should be applied to different countries. Countries with higher governance scores can be expected to respond faster to GAVI and other international vaccine initiatives. Although concerns regarding the financial sustainability of these immunization programs are expected, it is reasonable to assume that if financial resources are provided to nations with higher governance scores, vaccination programs that are in place will continue. However, in the absence of good country-level governance, such as in the case of country group III, the ability to respond to international vaccine initiatives will likely be slower and the optimal use of financial assistance may be at risk.

Although the countries that pioneered the Hib program in sub-Saharan Africa had overall good governance scores, a real question remains whether, absent some significant price concession, even they can sustain this expensive vaccine once GAVI funding ends. This question has become more relevant since GAVI has continued to approve Hib vaccine funding to additional African countries (www.gavialliance.org/media_centre/press_releases/2007_11_29_en_pr_hib_boost.php). While some of these newly approved countries had already introduced the HepB vaccine beforehand, and they belong to an overall more governmentally resourceful group of countries, most of them had not used Hib or HepB vaccine before, and they belong to an overall weaker group of countries with less effective governmental systems.

GAVI has already begun modifying its criteria for financial support for fragile countries. These include providing funds to strengthen health systems (www.gavialliance.org/performance/evaluation/index.php) and requiring lower co-pays for vaccines (http://hibaction.org/resources/hibfocus/061117_alert/) [14]. The need for additional support and different rules of engagement with those nations (many of which are post-conflict) have been discussed during GAVI's meetings (www.gavialliance.org/resources/19brd_FragileStates.pdf). Analysis of the second phase of GAVI's funding (from 2007 to 2010) will be required to evaluate the success of the new changes in affecting new vaccine introduction. Ultimately, established criteria, such as those used by the Center for Global Development to assess the long-term success of global health initiatives, namely: scale, importance, impact, duration and cost-effectiveness [12], will probably be most suitable for this purpose.

The issues discussed in this paper are of paramount importance for the continued introduction of new vaccines into developing countries as well as maintaining and sustaining immunization programs. In this regard, additional new licensed vaccines such as Pneumococcal, Rotavirus, and Human papillomavirus vaccines are planned to follow HepB and Hib vaccines (www.gavialliance.org/vision/policies/new_vaccines/adips/index.php; www.gavialliance.org/resources/FS_HPV_EN.pdf). If these vaccines are successfully introduced into poor nations, they will likely facilitate the introduction of novel vaccines to prevent tuberculosis, malaria and HIV/AIDS when available.
